# Case Report: Management of tibial fracture nonunion: a case study of treatment failure and lessons learned

**DOI:** 10.3389/fsurg.2026.1891731

**Published:** 2026-09-03

**Authors:** Chaoshuai Wang, Chen Wang

**Affiliations:** 1 The First Clinical Medical College, Shandong University of Traditional Chinese Medicine, Jinan City, China; 2 Department of Orthopedics, The Second Affiliated Hospital of Shandong University of Traditional Chinese Medicine, Jinan City, China

**Keywords:** case report, locking plate fracture, mechanical stability, platelet-rich plasma, tibial nonunion

## Abstract

Concept of “mechanical–biological balance” is fundamental to successful fracture healing; disruption of either component can lead to healing failure. Fractures of the middle and distal tibia are commonly encountered in clinical practice, particularly when associated with comminution. Managing fracture nonunion requires a comprehensive assessment of infection status, skeletal alignment, bone defect, implant stability, and host biological characteristics. This case report aims to explore the etiological factors of tibial nonunion and to reflect upon the biomechanical mechanisms underlying internal fixation failure. The findings demonstrate that failure to restore structural stability rendered subsequent biological repair measures ineffective.

**Case presentation:**

A 49-year-old perimenopausal woman developed tibial nonunion after internal fixation with a medial locking plate, screws, and titanium cables for a comminuted mid-to-distal tibiofibular fracture. Despite subsequent revision with PRP injection and autologous iliac crest bone grafting without addressing the underlying mechanical instability, the nonunion persisted and ultimately resulted in plate fatigue fracture

**Conclusion:**

This case underscores that mechanical stability is a prerequisite for the biological cascade of fracture healing. In the absence of an adequate mechanical environment, even the most advanced biological augmentation cannot ensure successful union.

## 1 Introduction

The distal one-third of the tibia, particularly in cases complicated by comminuted fractures, often presents significant challenges in clinical healing. For established fracture nonunion, conventional approaches typically involve autologous iliac bone grafting and platelet-rich plasma (PRP) therapy, with more invasive options such as revision internal fixation (utilizing external fixators or intramedullary nails) also considered. However, these methods are associated with considerable trauma and a heightened risk of complications. This case study aims to explore the causes of fracture nonunion, the limitations of current treatments, and the mechanisms underlying internal fixation failure through the analysis of a failed treatment case involving tibial fracture nonunion.

## 2 Case presentation

**Figure 1:**
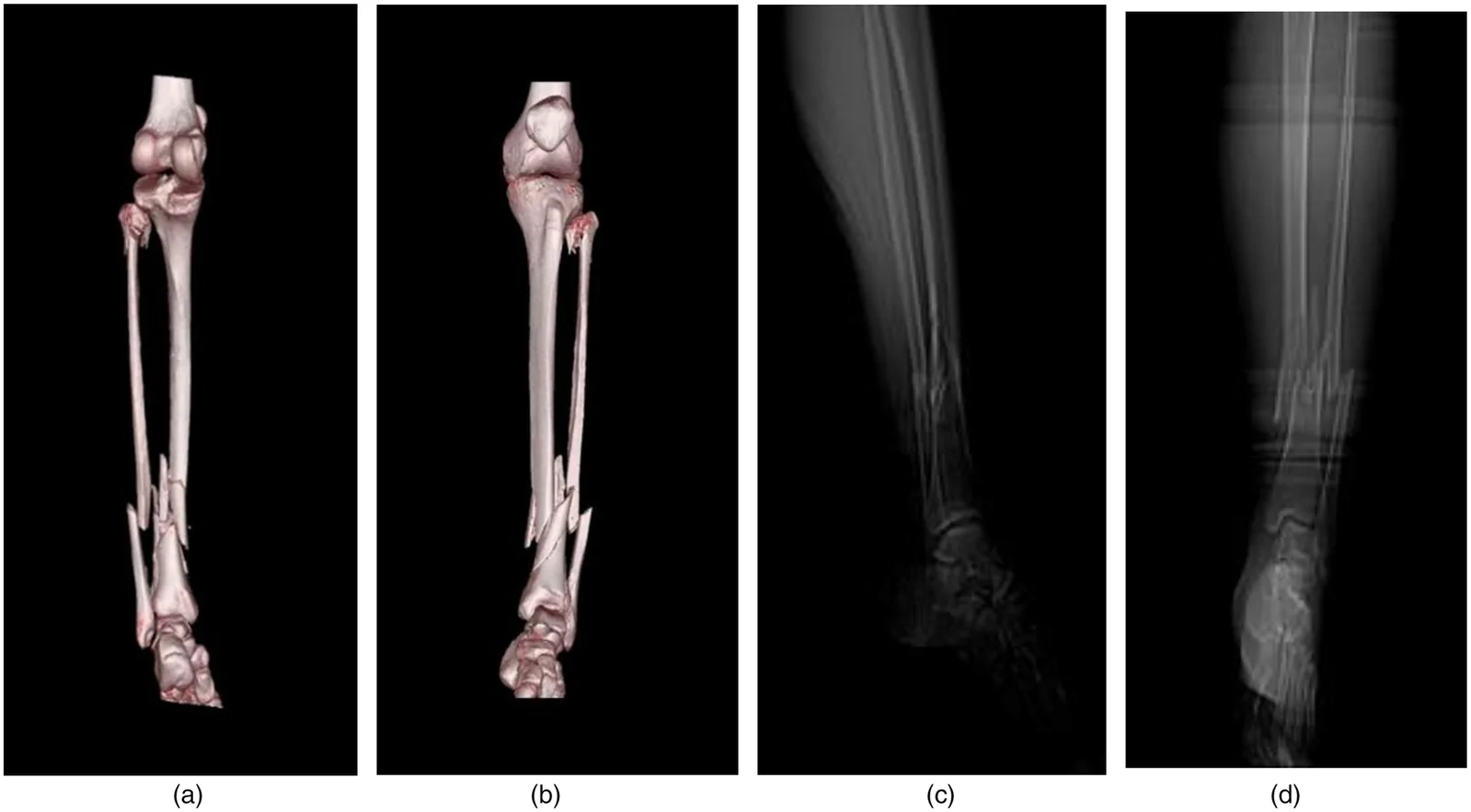
**(a–d)** on CT scans taken after admission, a comminuted fracture of the distal tibia is visible.

A 49-year-old female sustained an accidental crush injury to her left lower leg during work in September 2024, resulting in fractures of the middle and distal tibia as well as the distal fibula (Figures 1a–d). The fracture was classified as a closed, high-energy comminuted distal tibial fracture (AO/OTA type 43-A3). No associated neurological or vascular injuries were identified, and the surrounding soft tissues remained intact without skin laceration or signs of compartment syndrome. A detailed medical history revealed no diabetes mellitus, chronic kidney disease, inflammatory arthritis, long-term corticosteroid use, metabolic bone disease, or smoking.

**Figure 2:**
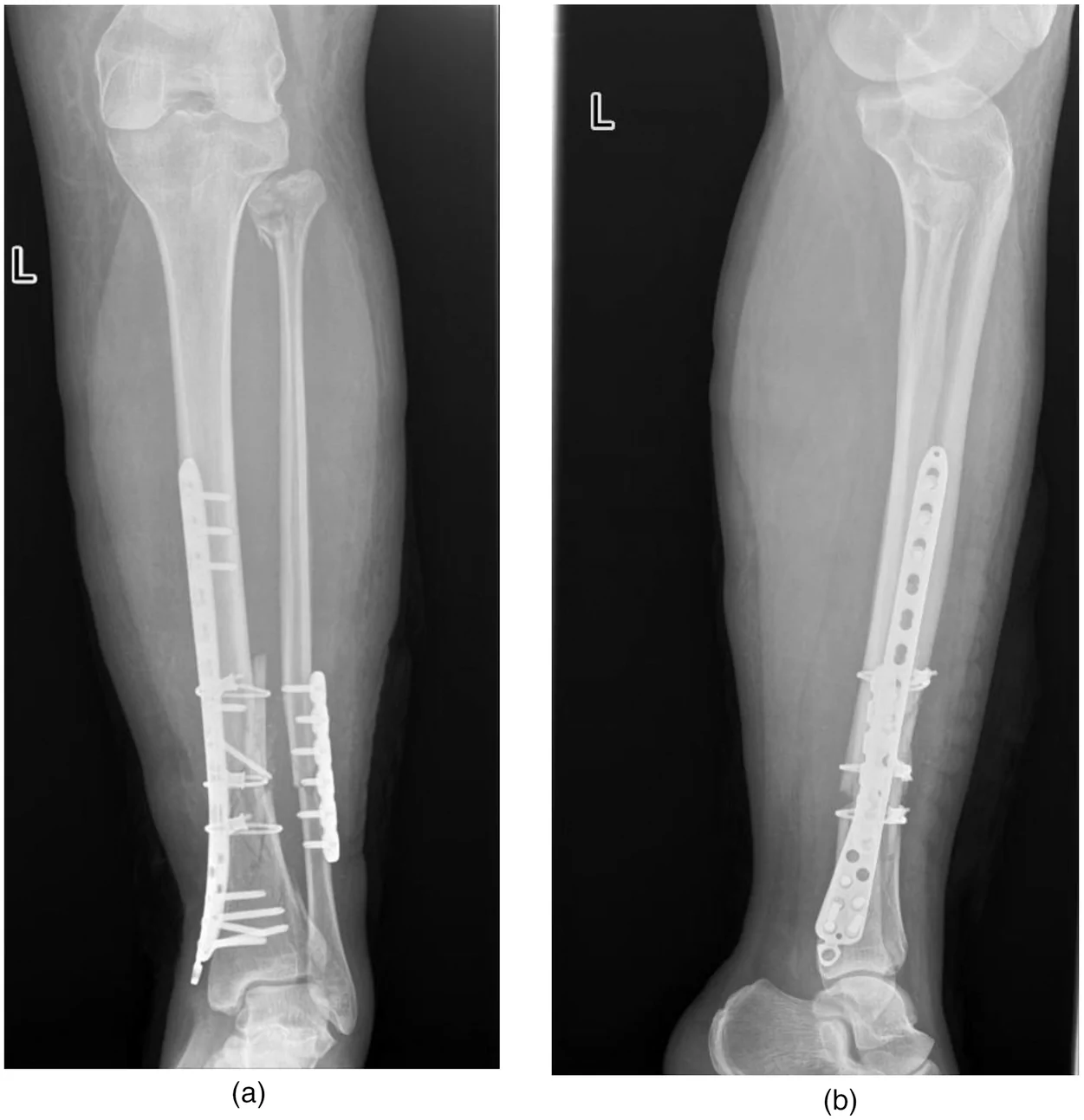
**(a,b)** on postoperative follow-up x-ray, satisfactory alignment of the fracture fragments is observed.

During the surgery, a 6-hole locking plate with 6 screws was utilized for fixation on the fibular side, while a single locking plate for the medial distal tibia, along with 9 screws, was applied on the tibial side. To prevent fragment displacement, three additional titanium cables were used for cerclage wiring to stabilize the bone fragments. Thereafter, 18 mL of allogeneic bone graft and 0.3 g of decalcified human dental matrix material were implanted around the defect to augment the bone stock and facilitate fracture healing. Decalcified human dental matrix was used as an osteoconductive graft substitute because of its mineralized collagen matrix and potential to provide a scaffold for bone regeneration (1). The patient was kept non-weight-bearing for 6 weeks postoperatively, followed by gradual progression to partial weight-bearing guided by clinical and radiographic evaluation. An x-ray re-examination on the first postoperative day is shown in Figures 2a,b. The fracture ends are well-apposed with stable disruption sites.

**Figure 3:**
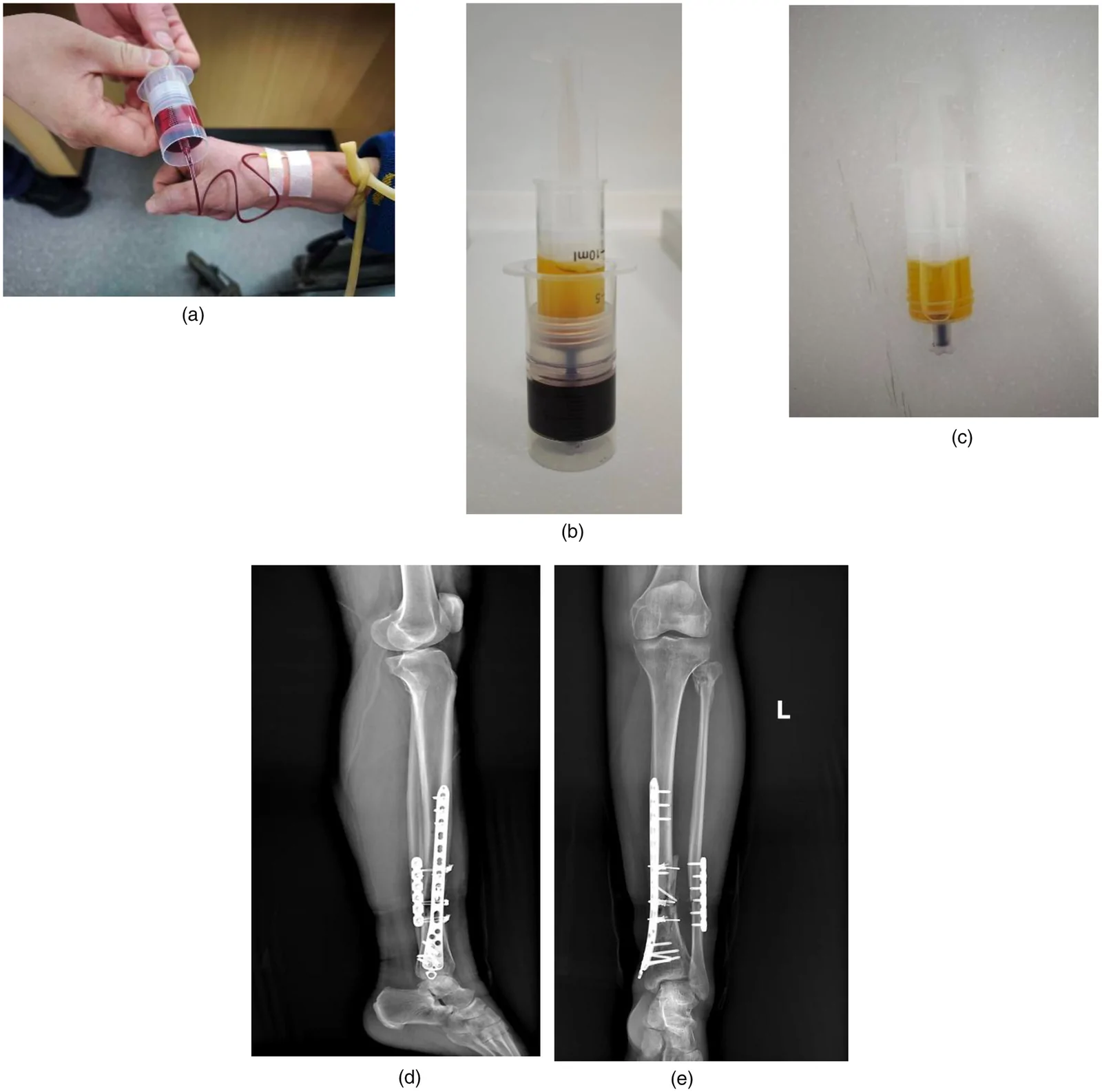
**(a–c)** depict the steps involved in PRP preparation. **(d,e)** No continuous callus bridging is observed at the tibial fracture site, with a distinct fracture line visible.

Upon x-ray re-examination in January 2025 (as shown in Figures 3d,e), Radiographic examination revealed an absence of continuous callus formation traversing the tibial fracture gap, and a distinct fracture line remained evident. Assessment using the RUST (Radiographic Union Scale for Tibial fractures) scoring system showed no appreciable evidence of progressive union. The patient reported no recent fever, local swelling, redness, heat, or pain indicative of infection. To promote fracture healing, the first Platelet-Rich Plasma (PRP) treatment was initiated in the outpatient setting. The PRP preparation process was as follows: Under sterile conditions, 40 mL of blood was drawn from the patient's elbow vein and centrifuged using a specialized kit to obtain approximately 5 mL of platelet-rich plasma. Under C-arm fluoroscopy guidance, PRP was injected through punctures at the anterior and lateral aspects of the tibia, as well as the lateral aspect of the fibula. The patient experienced no adverse reactions postoperatively (as shown in Figures 3a–c).

**Figure 4:**
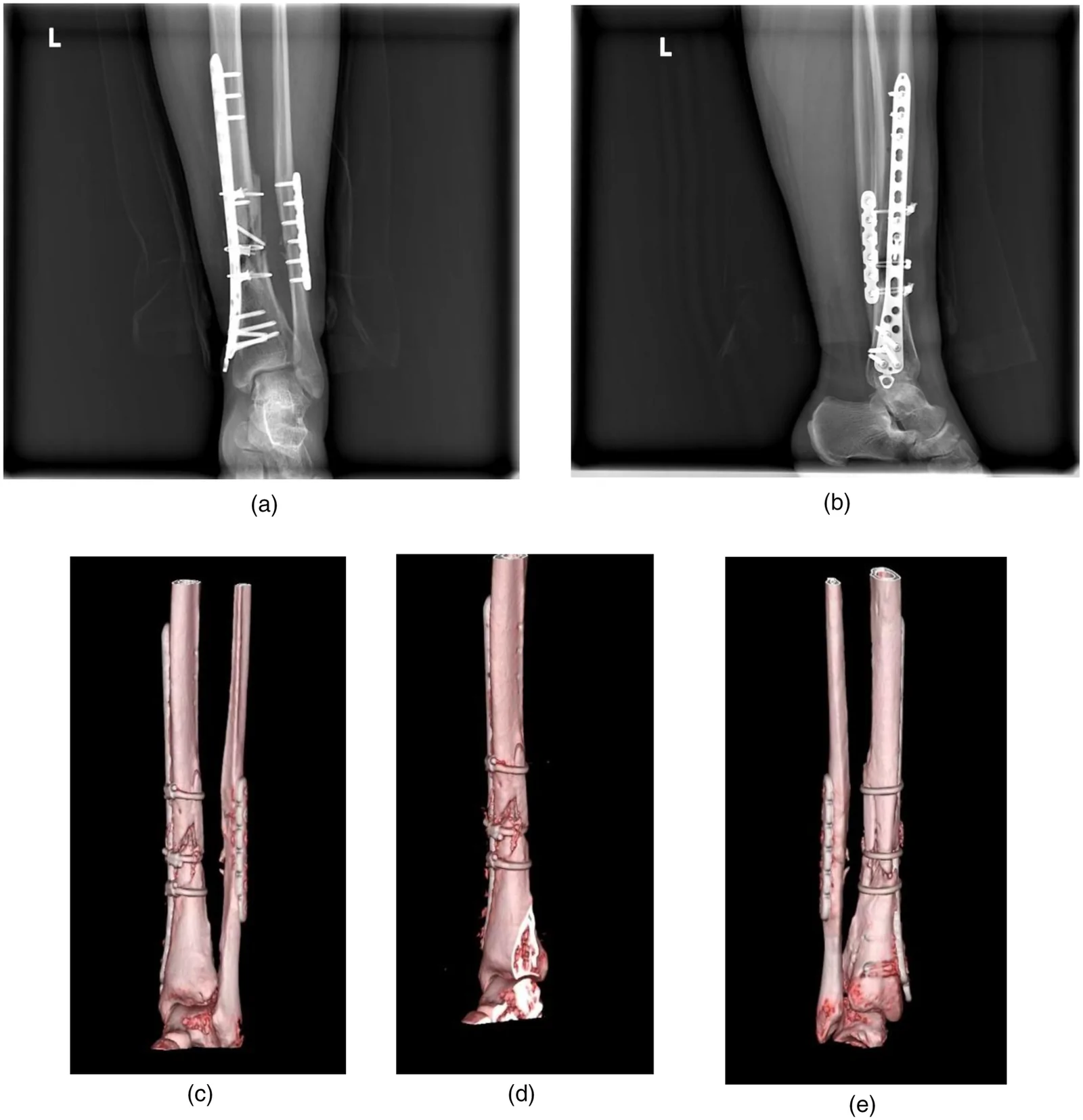
**(a,b)** on x-ray and **(c–e)** on CT, fracture healing at the tibial fracture site remains indistinct, while laboratory tests in Table 1 indicate no evidence of infection.

The patient was subsequently scheduled for PRP treatment at two-month intervals. By June 2025, after three PRP sessions, radiographic and CT evaluation (Figures 4a–e) demonstrated union of the fibula, whereas the tibial nonunion remained largely unchanged. The persistent lack of tibial healing raised concern regarding local mechanical factors, including stress shielding or mechanical interference from implants (particularly titanium cables and screws crossing the fracture gap), and revision surgery was therefore indicated.

Revision surgery was indicated. Preoperative inflammatory markers were within normal ranges (CRP: 1.8 mg/L; PCT: 0.038 ng/mL; Table 1), and the patient exhibited no clinical signs of infection, such as fever, wound drainage, erythema, or local hyperthermia. Intraoperative exposure of the fracture site revealed viable bone ends with satisfactory local blood supply, and no purulent discharge or malodor was observed. Based on the available clinical and laboratory findings, combined with the protracted duration of nonunion of approximately nine months, a preliminary working diagnosis of suspected aseptic nonunion was established (2). Nevertheless, the absence of intraoperative deep tissue cultures precluded definitive exclusion of low-grade infection.

**Table 1:** Preoperative inflammatory markers before revision surgery.

Marker	Value	Reference range
CRP	1.8 mg/L	< 5.0 mg/L
PCT	0.038 ng/mL	< 0.05 ng/mL

**Figure 5:**
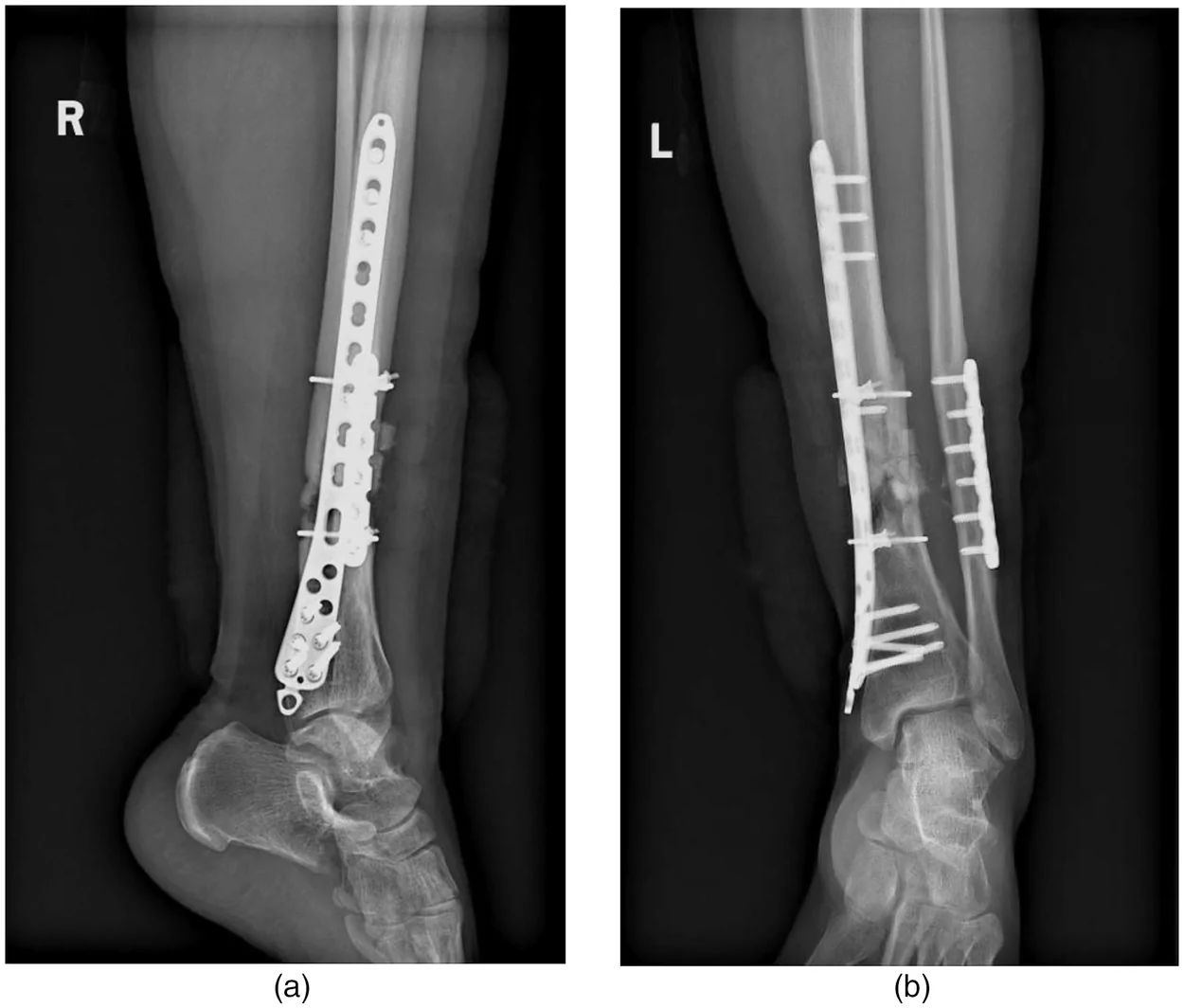
**(a,b)** on postoperative follow-up x-ray.

During the surgery, one screw and one titanium cable within the fracture - line region that might have impeded healing were removed, while the main plate was retained to minimize trauma. However, at that time, the issue of stress concentration resulting from the decreased fixation rigidity was not recognized. Subsequently, autologous cancellous bone was harvested from the iliac crest and grafted around the non - union site of the tibia. Postoperative x-rays (Figures 5a,b) demonstrated that the bone graft was in a good position. The patient was instructed to avoid weight - bearing and to continue receiving PRP treatment once a month.

**Figure 6:**
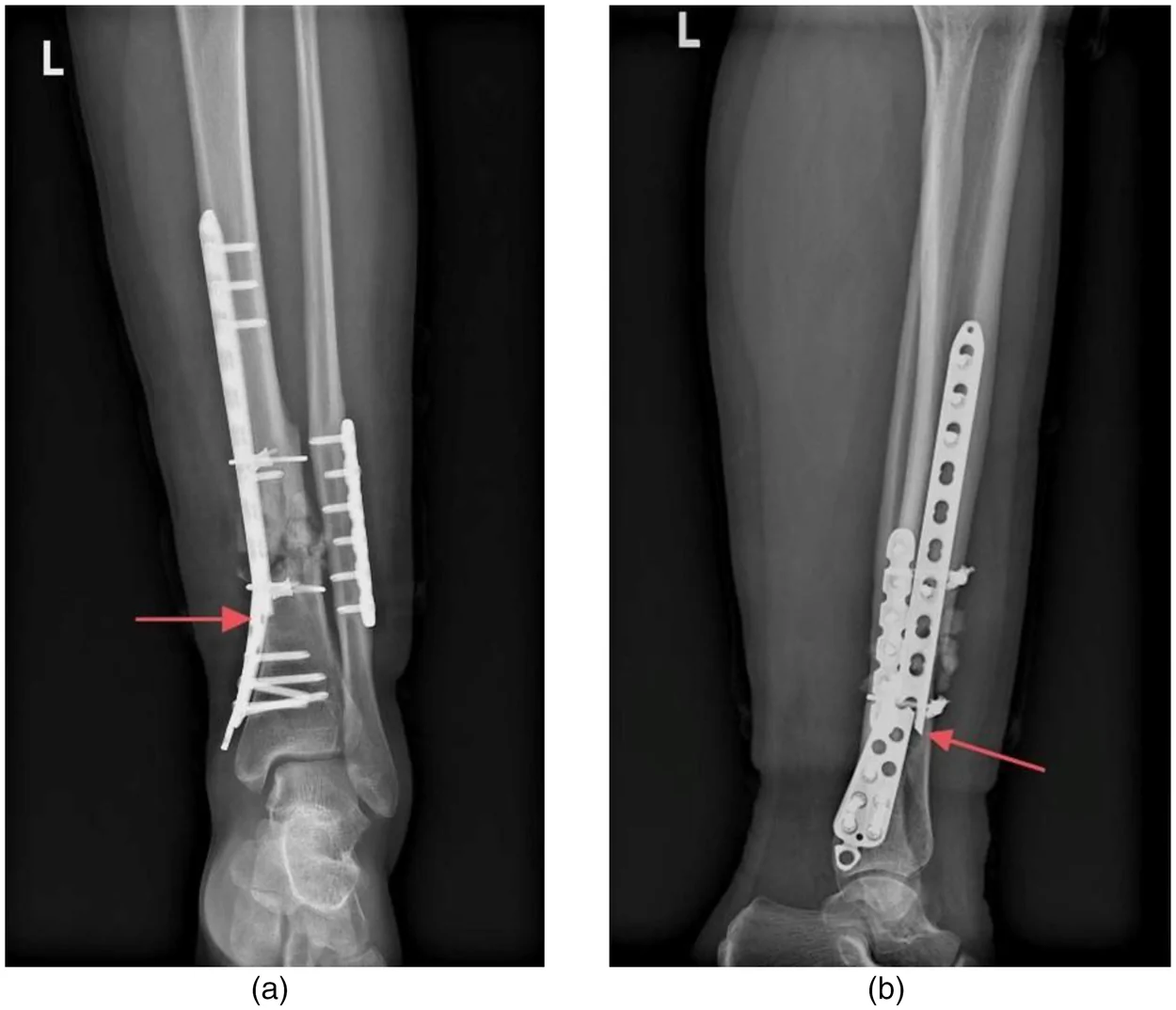
**(a,b)** on x-ray, plate fracture is observed with nonunion of the fracture site.

Following PRP treatment in September 2025, the patient underwent an x-ray re - examination, which revealed a complete fracture of the medial locking plate of the tibia at the site corresponding to the non - union gap, as shown in Figures 6a,b. At this time, the patient still had no clinical symptoms, but the imaging findings confirmed the ultimate failure of the treatment (Table 2).

**Table 2:** Summary timeline of clinical course.

Date	Intervention	Key radiographic finding
September 2024	Index surgery: medial tibial locking plate + screws + titanium cables; fibular plate; allograft + dental matrix graft	Postoperative x-ray: satisfactory alignment, fracture ends well-apposed (Figures 2a,b)
January 2025	First PRP injection (outpatient)	x-ray: no continuous callus, distinct fracture line (Figures 3d,e)
March 2025	Second PRP injection	—
May 2025	Third PRP injection	—
June 2025	Revision surgery: removal of one screw + one titanium cable; autologous iliac crest bone grafting	x-ray/CT: fibula united, tibial nonunion unchanged (Table 1); inflammatory markers normal
September 2025	Post-revision follow-up	x-ray: plate fatigue fracture at nonunion gap (Figures 6a,b)

## 3 Discussion

This case systematically illustrates the failure course of a patient with a tibial fracture, from the initial surgery in September 2024 to the complete breakage of internal fixation without fracture union by September 2025. Despite interventions including platelet-rich plasma (PRP) therapy, iliac bone grafting, and partial hardware removal, the outcomes remained unsatisfactory. Nevertheless, this case offers valuable insights into the etiology of fracture nonunion, the limitations of biological treatments, and the mechanisms underlying internal fixation failure.

## 4 Possible causes of nonunion (of bones)

The pathogenesis of nonunion is generally attributed to the interplay of systemic and local factors (3, 4). In this case, the patient was a perimenopausal woman of approximately 50 years of age, and the associated metabolic bone alterations may have had a modest impact on fracture healing. Nevertheless, in the absence of bone mineral density assessment and metabolic bone marker profiling, osteoporosis could not be substantiated. Local factors were therefore considered more influential in this instance, including the inherently precarious vascularity of the distal tibia (5), the soft tissue and perfusion disruption caused by a high-energy comminuted injury, and the sustained lack of mechanical stability. These elements collectively predisposed to the development of nonunion.

A further limitation of this case pertains to the absence of routine intraoperative microbiological tissue sampling during the revision surgery. Despite the lack of clinical signs of infection and normal serum inflammatory markers (CRP and PCT), low-grade fracture-related infection could not be definitively excluded. For future revision procedures in cases of suspected aseptic nonunion, multiple deep tissue specimens should be obtained for microbiological culture, as recommended by current guidelines for fracture-related infection, to improve diagnostic certainty.

## 5 Mechanisms underlying the limited efficacy of PRP and autologous iliac bone grafting

For the management of nonunion, platelet-rich plasma (PRP) and autologous iliac crest bone grafting are widely used therapeutic options (6). PRP is rich in growth factors and cytokines, which can promote the healing of osseous defects (7). In a study by Bielecki et al. (8), 13 of 20 patients with nonunion achieved uneventful healing at an average of 10.3 weeks following PRP treatment. Autologous iliac bone grafting, on the other hand, provides an intact osteogenic matrix and cellular components, creating a stable mechanical milieu conducive to bone healing (9–12).

Interestingly, despite multiple PRP treatments, the fibula achieved union, whereas the tibia remained ununited despite sharing the same systemic biological environment and the same treatment protocol. This differential healing pattern suggests that local mechanical conditions are more critical than systemic biological factors in determining healing outcomes.

This further demonstrates that the persistent lack of mechanical stability in this case compromised the efficacy of biological interventions in promoting fracture healing. This should not be interpreted as a refutation of the clinical utility of PRP therapy, but rather as an illustration that biological augmentation has substantial limitations when mechanical fixation is inadequate.

## 6 Biomechanical analysis of plate fracture

The revision strategy in this case was constrained by a significant decision-making limitation. Concern over extensive surgical exposure and additional soft tissue damage prompted the removal of only one screw and one titanium cable spanning the fracture zone, with the original locking plate left *in situ*. However, by the time of the revision—approximately nine months after the index procedure—the fracture had already shown no evidence of bony bridging on serial imaging. This means that from the early postoperative period onward, the plate had been the sole load-bearing structure across the nonunion gap, transmitting all physiological loads through the implant alone. Fatigue failure is a cumulative-damage process: under repetitive cyclic loading, fatigue cracks typically initiate on the tension side of the plate and propagate progressively over many loading cycles. The removal of fixation points across the nonunion site during the revision reduced the overall stiffness of the construct and created a local stress riser, which accelerated an already-advancing fatigue process. This ultimately culminated in complete plate breakage. Thus, the revision should be understood not as the origin of the fatigue failure, but as an exacerbating factor superimposed on a pre-existing cumulative mechanical burden.

Locking plates are intended to confer adequate mechanical stability to the affected extremity and to support osseous healing at the fracture site (13). The process of implant failure follows a well-recognized sequence: crack initiation, fatigue crack propagation, and catastrophic fracture (14). In this case, there was no bony continuity at the fracture ends, and all weight-bearing stress was borne solely by the unilateral plate. Under prolonged cyclic loading, fatigue cracks gradually developed on the tension side of the plate and propagated progressively, ultimately resulting in the complete failure of the revision treatment.

## 7 Considerations on the selection of surgical fixation methods

Despite acceptable reduction and alignment following the initial osteosynthesis, a retrospective review indicates that several factors jointly established a mechanical and biological milieu that was suboptimal for fracture healing. In accordance with AO Trauma principles, the surgical management of comminuted distal tibial fractures necessitates a careful equilibrium between adequate mechanical stability and preservation of local biological activity at the fracture site (15, 16).

In this case, long-span bridge plating, cerclage cables, and screws placed near the fracture zone may have adversely affected biomechanics and blood supply, contributing to nonunion (17). In the revision surgery, only partial hardware removal was performed, leaving the original fixation in place, based on the expectation that biological stimulation would promote healing without added trauma. This approach underestimated persistent mechanical instability. A more comprehensive revision—replacing failed implants, restoring stability, and adding biological augmentation—might have achieved better healing. This experience shows that tibial nonunion treatment requires concurrent mechanical and biological restoration, not biological enhancement alone.

In hindsight, this approach likely underestimated the consequences of ongoing mechanical instability. A combined construct incorporating both intramedullary nailing and plate fixation—although entailing more extensive surgical exposure—might have offered distinct advantages: the intramedullary nail would have provided central axial support with more evenly distributed loading across the limb, while the plate would have served as local augmentation to address the inherent limitations of nail-alone fixation in the presence of comminuted fragments (18, 19).

## 8 Overall context of complex lower extremity reconstruction surgery

The treatment of tibial fractures should be framed within the broader paradigm of complex lower extremity reconstruction. High-energy trauma—such as knee dislocations or tibial fractures—carries a particularly challenging prognosis, as these injuries frequently involve bone loss, soft tissue compromise, vascular injury, and infectious risk (20, 21). Under such circumstances, fracture healing is governed by a multitude of factors, including mechanical stability, vascular perfusion, bone defect size, infection status, host biology, and soft tissue integrity (22). For post-traumatic or post-debridement bone defects, a spectrum of reconstructive strategies must be carefully deliberated, including bone grafting, the induced membrane technique, and distraction osteogenesis. Adjunctive measures—such as PRP, hyperbaric oxygen, teriparatide, bone grafting, or revisional surgery—may facilitate healing (23, 24); however, these should be viewed as adjuncts to, not replacements for, standard care. Accordingly, the reconstructive algorithm should adhere to a disciplined sequence: exclusion of infection (with intraoperative tissue sampling and culture), debridement as necessary, restoration of alignment and stability, management of bone defects, and finally, directed biological augmentation (25, 26).

## 9 Conclusion

This case underscores that a stable mechanical environment is foundational to successful bone healing in nonunion management. A sequential algorithmic approach—"exclude infection, restore mechanical stability, then apply biological augmentation"—should be adopted, with tailored decisions informed by bone defect length, soft tissue envelope, and mechanical axis alignment. The operative sequence may encompass debridement (resection of nonviable tissue), revision osteosynthesis (via intramedullary nailing, double-plating, or combined nail-plate constructs), followed by biological enhancement. This case suggests that, in similar clinical scenarios, restoration of mechanical stability should be considered before biological augmentation during revision procedures.

## Data Availability

The original contributions presented in the study are included in the article. Further inquiries can be directed to the corresponding author.
